# Neurological complications associated with rapid weight loss and nutritional deficiencies following GLP-1 agonist use: a case report

**DOI:** 10.1186/s12883-025-04540-7

**Published:** 2025-11-26

**Authors:** Ali Zahir, David Collins, Seyvonne Ip, Peyman Samghabadi, Vanja C. Douglas, Sara C. LaHue

**Affiliations:** 1 Department of Neurology, School of Medicine, University of California, San Francisco, USA; 2 UCSF Weill Institute for Neurosciences, Department of Neurology, University of California, San Francisco, CA USA; 3 Department of Medicine, School of Medicine, University of California, San Francisco, USA; 4 Department of Pathology, School of Medicine, University of California, San Francisco, USA

## Abstract

GLP-1 agonists have been a welcome addition to the armamentarium in the treatment of diabetes and obesity. While this class of medications is generally considered safe and effective, potentially severe neurological complications may be associated with rapid weight loss and nutritional deficiencies following GLP-1 agonist use. We present a 37-year-old woman who was prescribed semaglutide for diabetes and weight loss who subsequently experienced significant weight reduction and rapid glycemic control over three months. Thereafter, she developed progressive right leg numbness and weakness, followed by similar symptoms in the left leg, and blurred vision. Initially diagnosed with B12 deficiency, her symptoms worsened despite supplementation, leading to acute encephalopathy and transfer to a tertiary center. Neurological examination revealed disorientation, ocular abnormalities, weakness, sensory deficits, and preserved ankle jerks. Diagnostic workup was notable for thiamine deficiency, NCS/EMG showing a severe, axonal polyneuropathy, and nerve biopsy redemonstrating severe axonal neuropathy. After extensive diagnostic workup, the most likely etiology of her clinical presentation was favored to be non-alcoholic Wernicke’s encephalopathy (NAWE) and treatment-induced neuropathy of diabetes (TIND). This case suggests that vulnerable individuals may experience significant adverse neurological complications from the metabolic effects of GLP-1 agonist use. As a class effect, the benefits of these medications outweigh the risks, but their use warrants consideration of their potential sequelae.

**Supplementary Information:**

The online version contains supplementary material available at 10.1186/s12883-025-04540-7.

## Background

GLP-1 receptor agonists have transformed the therapeutic landscape for both type 2 diabetes mellitus and obesity, primarily by modulating appetite and gastric emptying to facilitate weight loss [1]. While these medications offer significant metabolic benefits, the physiological alterations they can induce are not without potential concerns. This case report serves to highlight a critical, albeit rare, association between GLP-1 agonist therapy and the subsequent development of two distinct neurological complications: non-alcoholic Wernicke’s encephalopathy (NAWE) and treatment-induced neuropathy of diabetes (TIND). The emergence of NAWE in this patient underscores the importance of considering thiamine deficiency as a potential consequence of GLP-1 agonist-induced appetite suppression and altered nutrient intake or absorption. Simultaneously, the development of TIND suggests that the rapid improvements in glycemic control often achieved with these agents can paradoxically trigger neuropathic syndromes in some individuals. This unique presentation of both NAWE and TIND in a single patient following GLP-1 agonist use warrants careful consideration of the potential central and peripheral neurological sequelae of these increasingly prevalent medications and emphasizes the need for heightened clinical awareness and appropriate monitoring strategies.

## Case report

A 37-year-old woman with hypertension, chronic kidney disease, hypothyroidism, diabetic retinopathy, and adult-onset diabetes mellitus presented with four months of progressive weakness and numbness. She began semaglutide 0.5 mg weekly three months prior to the symptom onset for weight loss and management of diabetes. This resulted in recurrent emesis and low appetite, with a consequent 45-kilogram (100 lbs) weight loss from a baseline of 158.8 kg (350 lbs), and a seven-point reduction in glycated hemoglobin from a baseline of 14% over the course of ten weeks. Her initial symptom was proximal right leg numbness, followed by sharp pain and then proximal right leg weakness in the following days, which all spread distally over the course of two weeks. Four weeks after the onset of her right leg symptoms, she began experiencing similar symptoms in the left leg with a similar tempo and developed bilateral blurry vision several days after the onset of her left leg symptoms, and thereafter presented to a local hospital due to blurry vision. Prior to this episode, she had no reported neuropathy symptoms and exhibited full functional independence. At the local hospital, she was reported to be diagnosed with B12 deficiency (lab value unavailable for our review), and started on oral cobalamin supplementation, and discharged with outpatient follow-up. In the following weeks, the weakness did not improve, and she began experiencing similar numbness followed by pain and weakness in her bilateral upper extremities as well as urinary retention. Acute confusion prompted her family to bring her into a local hospital, where she was then admitted, and shortly thereafter, she was transferred to our tertiary care center. Her other medications at the time of local hospital admission included insulin, metformin, glipizide, levothyroxine, hydrocodone, and Cobalamin. Her neurological examination at our hospital was notable for disorientation to date and inability to state months of the year backward; saccadic intrusions on smooth pursuit and both hypometric and hypermetric saccades; bilateral pronator drift more prominent in the left arm; diffuse bilateral arm weakness that was worse in the left [(Medical Research Council [MRC] Scale: deltoid 4-, triceps 4, biceps 4+, extensor carpi radialis 4+, flexor carpi radialis 4+, extensor digitorum communis 4+, flexor digitorum superficialis 5)]. There was flaccid lower extremity paraplegia with MRC grading of 0. Bulk was normal. Her reflexes were absent at the knees and 2+ at the biceps, triceps, finger flexors, and ankles. There was reduced sensation to light touch, vibration, and pinprick in the distal legs and hands with allodynia in that same distribution; joint position sense was absent in the toes and ankles and reduced in the fingers. There was ataxia on finger-nose-finger bilaterally. Ophthalmologic examination demonstrated diabetic retinopathy.

The patient’s laboratory workup was notable for the following (abnormal values provided with normal ranges in parentheses): a microcytic anemia (Hemoglobin 8.4 g/dL (12.0–15.5.0.5) with MCV 87 (80–100)); a mild cholestatic transaminitis (Alkaline phosphatase 193 U/L (38–108); ALT 72 U/L (10–61); AST 66 U/L (5–44)); elevated hemoglobin A1C (A1C: 6.7% (< 5.7%)); elevated inflammatory markers (C-reactive protein 49 mg/L (< 5.1); Erythrocyte sedimentation rate 100 mm/h (0–15)); elevated clotting factors (Fibrin D-Dimer: 631ng/mL (< 500); Fibrinogen 519 mg/dL (202–430)), nephrotic range proteinuria attributed to diabetic nephropathy (Protein/Creatinine Ratio, Urine: 5, 591 mg/g urea) and the following micronutrient deficiencies: Vitamin B1 61 nmol/L (74–222); Vitamin D 1–25 6ng/mL (20–40); Folate 2.5 ng/mL (> 2.6). In addition to the following positive serology tests: positive ANA (1:160); B2 microglobulin 5.74 mg/L (< 2.74); Anti-Cardiolipin Antibody IgG: 49.2 CU (< 20.1), Beta 2 glycoprotein IgG: 251.2 CU (< 20.1). Negative or normal serum tests included coagulation panel Vitamin B3, Vitamin B12, methylmalonic acid, homocysteine, lead, mercury, thallium, arsenic, HIV, RPR hepatitis B/C serologies, Lyme, Bartonella, SPEP/UPEP, rheumatoid factor, anti-double-stranded DNA antibodies, anti-neutrophil cytoplasmic antibodies, anti-citric citrullinated peptide antibodies, anti-gliadin antibodies, anti-endomysial antibodies, cryoglobulins, anti-smith antibodies, anti-ribonucleoprotein antibodies, Sjogren’s antibodies, IgG subclasses, IgE, complement C3 and C4 levels, angiotensin-converting enzyme, mitochondrial antibodies, GAD-65 autoantibodies, anti-GM1/2 antibodies, GQ1B antibodies, and Gd1a/b antibodies. Cerebrospinal fluid was unremarkable (WBC: 0, Protein: 37, Glucose: 91, IgG index: 0.55, zero oligoclonal bands); and microbiology cultures were negative.

**Fig. 1:**
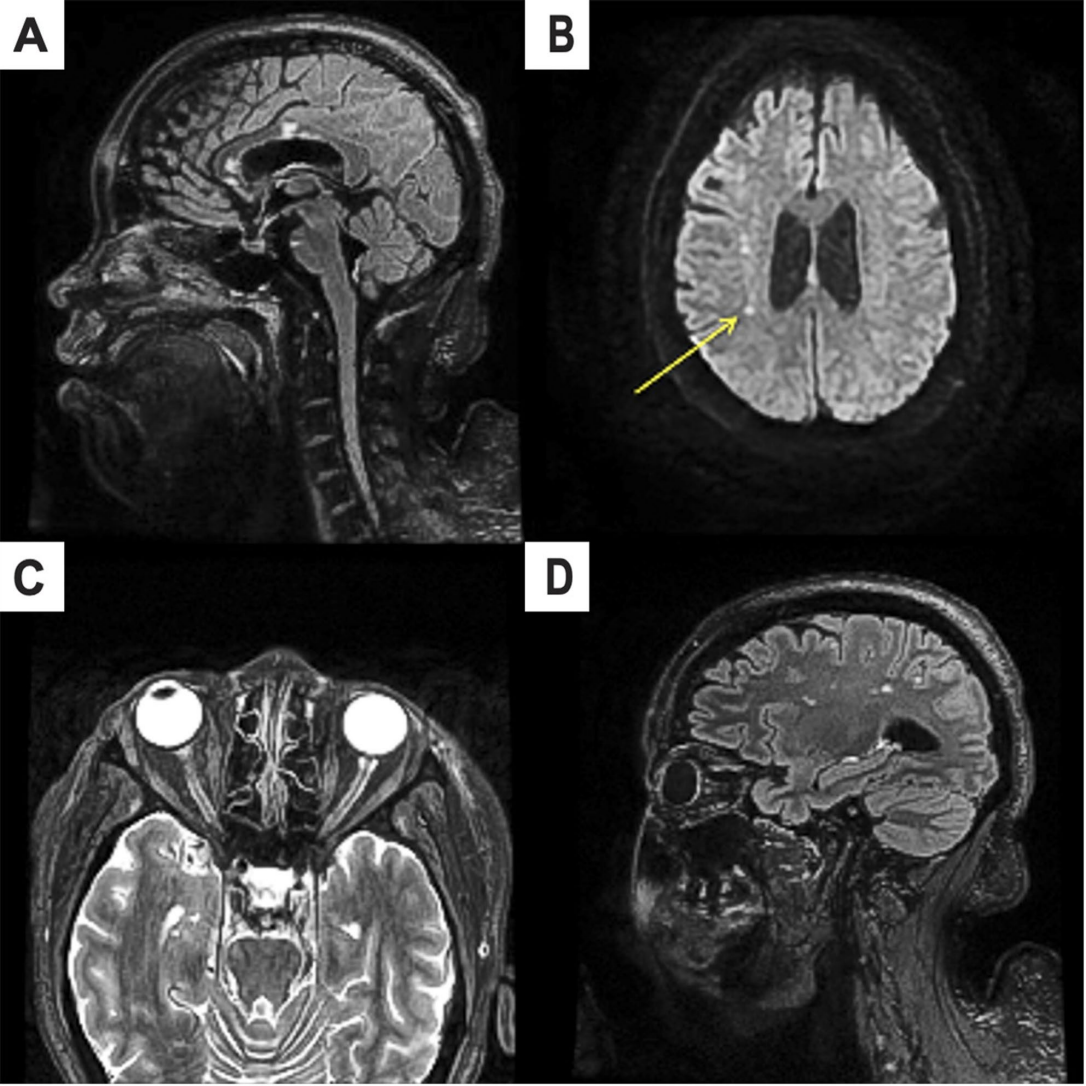
MRI brain and orbits with and without contrast findings. (**A**) T2 fluid-attenuated inversion recovery (FLAIR) hyperintensities along the right genu and body of the corpus callosum and ventral pons. (**B**) Diffusion restriction involving right corona radiata (**C**) MRI orbit with and without contrast demonstrating bilateral optic atrophy. (**D**) T2 FLAIR sagittal view redemonstrating lesions within right corona radiata

**Fig. 2:**
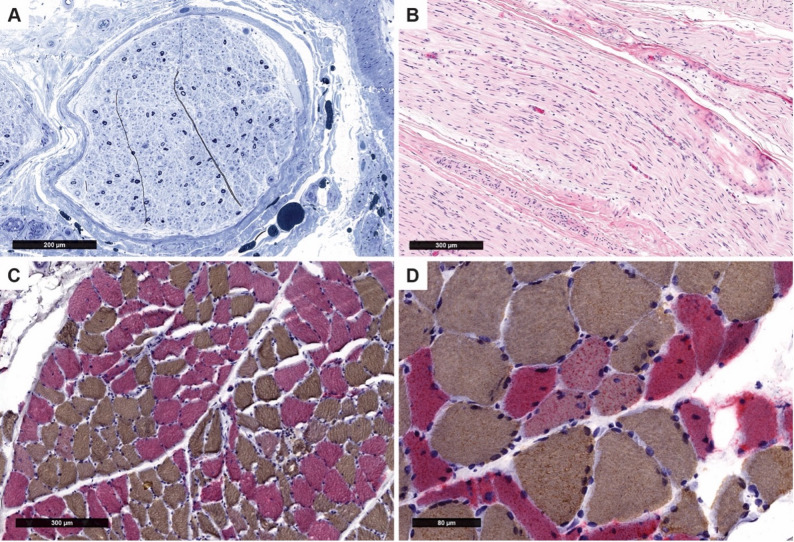
Sural nerve and gastrocnemius muscle biopsy findings. Left sural nerve: (**A**) Toluidine blue-stained section of Epon embedded material showing severe, diffuse loss of small and large axons with relative preservation of the latter. (**B**) Formalin-fixed paraffin-embedded (FFPE) H&E-stained section showing lack of significant inflammation or another acute process. Left gastrocnemius: (**C**) Cryosection stained with fast and slow myosin (dual myosin) immunohistochemistry showing scattered type 1 (brown chromogen) and type 2 groups (red chromogen). Scattered smaller, angular fibers. (**D**) Rare myofibers that express both myosin proteins

MRI of the brain and orbits with and without contrast (Fig. 1) showed diffusion restriction involving the right corpus callosum and right corona radiata with corresponding T2/FLAIR hyperintensities and additional foci of T2 hyperintensities without diffusion restriction involving the ventral pons in addition to atrophy of the bilateral optic nerves with no contrast enhancement. An MRI of the total spine with and without contrast was unremarkable. A digital subtraction angiogram of the head, chest, abdomen, and pelvis was unremarkable. A transthoracic echocardiogram and duplex ultrasound of the extremities did not reveal a source of embolism. She underwent inpatient electromyogram/nerve conduction studies (EMG/NCS) to evaluate for a demyelinating process. The results of the NCS and EMG (please refer to Supplementary Table 1 for EMG/NCS numeric values) were most consistent with a severe, diffuse, sensorimotor axonal polyradiculoneuropathy of the bilateral upper and lower extremities. Given the severe, atypical, and progressive neuropathy with uncertain etiology and EMG findings not fully consistent with demyelinating or vasculitic features, a sural nerve and muscle biopsy were performed. A biopsy of the left sural nerve and left gastrocnemius muscle demonstrated severe axonal neuropathy (Fig. 2). Visual evoked potentials were not performed.

Given the patient’s history and physical examination, coupled with a low vitamin B1 level and rapid correction in glycemic control, the most likely explanation for this patient’s presentation was thiamine deficiency and concurrent lumbosacral polyradiculopathy associated with treatment-induced diabetic neuropathy (TIND). Additionally, following discussion at a multidisciplinary neuroradiology conference, the MRI brain lesions observed on her brain imaging were most consistent with small vessel infarcts. Given her positive cardiolipin and beta-2-glycoprotein antibodies coupled with a clinical history of multiple unexplained spontaneous abortions and lack of embolic source, antiphospholipid antibody syndrome (APLS) was thought to be the most plausible explanation of the MRI findings. Besides thrombosis, APLS can affect the central nervous system through inflammation of the CSF and the peripheral nervous system through vasculitis, though neither was present here. Additionally, B12 deficiency is associated with increased thrombotic risk mediated by hyperhomocysteinemia. While MMA and homocysteine were not evaluated when the patient was first diagnosed with B12 deficiency, it is possible the vitamin deficiency unmasked subclinical APLS, which could explain the temporal coincidence of the patient’s strokes and other neurological symptoms. Notably, her thrombi would not be sufficient to explain her clinical presentation, and the location of the patient’s strokes was not sufficient to explain the encephalopathy, ocular dysmetria, or ataxia.

The patient was treated with thiamine, with improvement in mental status, ocular dysmetria, and ataxia, further supporting the diagnosis of thiamine deficiency. She was treated with warfarin for APLS, without progression of her cerebral lesions on follow-up imaging obtained one month after the initial scans. Six months after her initial presentation, her weakness continued to persist, and the patient remained primarily bedbound. Follow-up MRI Brain and orbit MRI obtained at that time did not show progression. She continued to have debilitating neuropathic pain that necessitated a complex pain regimen, including daily opioids.

## Discussion

The patient in this case report presented with a complex constellation of neurological symptoms, including progressive limb weakness, sensory disturbances, blurred vision, and acute encephalopathy, against a background of multiple chronic medical conditions and recent significant weight loss associated with semaglutide use. This patient’s illness evolved sub-acutely over several months and was accompanied by profound weight loss. Her neurological examination suggested multiple simultaneous localizations; therefore, a broad workup was sent for this patient. When contextualizing her examination findings within the diagnostic workup, the limb ataxia can be explained by diminished proprioception or cerebellar impairment as seen in Wernicke’s encephalopathy, while ocular dysmetria can also be seen in thiamine deficiency. The patient’s cognitive deficits can also be attributed to Wernicke’s encephalopathy, especially since her MRI lesions would not be expected to cause cognitive deficits. Often, patients with thiamine deficiency have concomitant nutritional deficiencies (such as B12 in this case), which can contribute to additional neurological symptoms and signs, such as optic atrophy in this case [1]. Her urinary retention could be secondary to dysautonomia, which is common in patients with TIND.

Building on the understanding that GLP-1 agonists induce weight loss through appetite suppression and slowed gastric emptying, it is crucial to recognize the potential downstream consequences of these mechanisms, particularly regarding nutritional status. While reduced oral intake is the primary driver of weight loss, delayed food transit through the digestive system can impact the absorption of essential macro- and micronutrients [2]. This altered absorption, coupled with a potentially less varied diet due to reduced appetite, creates a scenario where patients on GLP-1 agonists, especially those on higher doses or for prolonged durations, might be at an increased risk of developing nutritional deficiencies. Thiamine deficiency is characterized by confusion, oculomotor abnormalities, and difficulty walking, all of which were observed in this patient. Due to its necessity in myelin sheath maintenance, thiamine deficiency may also cause peripheral sensorimotor neuropathy [3]. Early detection and treatment can improve the prognosis, but neuronal damage can be irreversible [4]. A deficiency can, therefore, lead not only to the central nervous system manifestations seen in Wernicke encephalopathy but also to peripheral sensorimotor neuropathy, characterized by weakness, numbness, and pain in the extremities. Though a B12 deficiency can explain some of her findings, this patient continued to worsen despite B12 supplementation, and normalization of her B12 level suggests her presentation cannot be entirely attributed to vitamin B12 deficiency. Patients who are identified as having a nutritional deficiency should have a comprehensive nutritional screening coupled with early treatment, with a low threshold to supplement thiamine in addition to B12.

Treatment-induced neuropathy of diabetes (TIND) is an uncommon yet debilitating complication following rapid improvement in glycemic control [5, 6]. Diagnostics primarily rely on the temporal association of severe neuropathic pain and/or motor weakness with a rapid and substantial improvement in glycemic control in patients with pre-existing diabetes. Dysautonomia is another common clinical finding. In our patient, urinary retention may have been secondary to dysautonomia as a result of TIND; however, it is difficult to prove given other evidence of dysautonomia on examination. Exclusion of other causes of neuropathy is crucial for diagnosis, as there are no specific pathognomonic tests for TIND. Notably, a substantial reduction in HbA1c exceeding 4% points within a 3-month timeframe is associated with a significantly elevated (80%) absolute risk of developing TIND [5]. GLP-1 receptor agonists are among the most effective non-insulin medications for lowering hemoglobin A1c (HbA1c) and often demonstrate a greater reduction in HbA1c compared to other classes of diabetes medications [5].

Clinically, TIND can manifest with prominent lower extremity weakness while characteristically preserving ankle reflexes, and it has also been reported to produce diabetic lumbosacral radiculoplexus neuropathy characterized by painful asymmetric weakness, findings that parallel the presentation observed in this patient [7–9]. Recent literature has associated the use of GLP-1 receptor agonists with an increased likelihood of developing diabetic lumbosacral radiculoplexus neuropathy (DLRPN) and common fibular neuropathy (CFN), with a recently published case-control study on this topic indicating a 51% and 30% increased risk, respectively [9]. This association appears to be linked to the rapid metabolic changes and weight loss induced by these medications [10].

Given the lack of specific disease-modifying treatments for TIND, symptomatic management becomes paramount. This often involves a multidisciplinary approach, utilizing various pharmacological agents to address the severe neuropathic pain in addition to physical therapy and occupational therapy to regain mobility, strength, and functional independence during the recovery phase. However, a considerable proportion of affected individuals experience a protracted recovery [11, 12], highlighting the potential for significant and prolonged morbidity.

While no features on the biopsy suggested microvasculitis, it remains a possible mechanism for the patient’s neuropathy, and its absence in the sample doesn’t entirely rule it out due to the possibility of sampling error. Prior literature has established the diagnostic importance of identifying microvasculitis in both DLRPN and TIND, and the need for thorough evaluation as finding microvasculitis may offer both diagnostic and therapeutic utility [13]. The current lack of a clear understanding of the precise pathophysiological mechanisms driving TIND further complicates management and highlights the urgent need for dedicated research efforts. Investigating potential contributors such as microvascular injury, inflammatory processes, alterations in nerve metabolism, and the role of specific glycemic control strategies is crucial for unraveling the etiology of TIND. Such research would not only pave the way for the development of effective preventative strategies, potentially identifying individuals at higher risk and informing safer approaches to glycemic management, but also for the discovery of targeted treatment modalities that could accelerate recovery and reduce the long-term burden of this debilitating complication, particularly considering our patient did not improve with supportive care. As described above, prior literature has demonstrated the role of microvasculitis in TIND; however, immunotherapy at this time is not standard of care. Given the widespread use of GLP-1 agonists for both on and off-label uses, increased awareness of TIND among clinicians is essential for prompt recognition, accurate diagnosis, and the initiation of treatment to mitigate the potential for significant and prolonged morbidity. Moreover, TIND can be misdiagnosed as other metabolic or neuropathic conditions, including diabetic polyneuropathy or Guillain-Barré syndrome, underscoring the importance of maintaining a high index of suspicion and developing clearer diagnostic criteria.

This patient’s case is a cautionary tale regarding the potential neurological complications of GLP-1 agonists. The literature on this class of medications primarily highlights that they are generally well-tolerated with a low incidence of significant neurological adverse events. Prior literature, particularly one other case report, has raised the possibility of an association between GLP-1 agonist use and the development of non-alcoholic Wernicke’s encephalopathy (NAWE), like our patient [14]. Thus, prior to prescribing these medications, clinicians should consider evaluation for pre-existing nutritional deficiencies through laboratory testing and dietary history and inquire about food security, as socioeconomic factors can significantly influence a patient’s ability to maintain a balanced diet while experiencing appetite suppression, as recommended by other authors [15]. Therefore, comprehensive counseling should be provided to patients so that they understand the mechanisms of action of GLP-1 agonists, the potential for weight loss and its associated benefits, and the possible unintended consequences. Although GLP-1 agonists represent a significant step forward in the treatment of diabetes and obesity, the potential risk of complications warrants careful monitoring and further investigation.

## Supplementary Information

Supplementary Material 1.

## Data Availability

All data are included in the electronic medical record of the patient. The clinical data from this case report are not publicly available due to our policy statement of sharing clinical data only on request, but are available from the corresponding author on reasonable request.

## Abbreviations

GLP: Glucagon Like Peptide
NAWE: Non Alcoholic Wernicke’s encephalopathy
TIND: Treatment Induced Neuropathy of Diabetes
