# Supplementary material for: Neurological complications associated with rapid weight loss and nutritional deficiencies following GLP-1 agonist use: a case report
Source: BMC Neurol. 2025 Nov 26;26:5. doi: 10.1186/s12883-025-04540-7 (PMC12764084; doi:10.1186/s12883-025-04540-7)
Supplement: Supplementary file 1 — Supplementary Material 1. [file 12883_2025_4540_MOESM1_ESM.docx]

**Normal Values** **ANTIDROMIC SENSORY MOTOR**

Nerve Conduction Amplitude **AGE** Nerve Dist Lat Conduction Velocity Amplitude

Velocity (m/s) (m V) (years) (ms) forearm/leg elbow/knee upper arm (mV)

Median (W-D2) 45 15 5-40 Median (APB) 4.2 50 - 52 5.0

Ulnar (W-D5) 45 12 5-40 Ulnar (ADM) 3.7 50 42 52 5.0

Sural 42 6 5-40 Tibial (AH) 6.5 41 - - 3.0

Superf Peroneal 42 4 5-40 Peroneal (EDB) 6.0 41 38 - 2.0

Lower limits for nerve conduction studies set at 33° C for arm and 32° C for leg.

**SNC**

| **Nerve / Sites** | **Rec. Site** | **Onset Lat** | **Peak Lat** | **NP Amp** | **Segments** | **Distance** | **Velocity** | **Temp.** | **Stim. Dur** |
| --- | --- | --- | --- | --- | --- | --- | --- | --- | --- |
|  |  | **ms** | **ms** | **µV** |  | **mm** | **m/s** | **°C** | **ms** |
| **R Median - Digit II (Antidromic)** | | | | | | | | | |
| Wrist | Dig II | 3.0 | 3.8 | **5.1** | Wrist - Dig II | 140 | 47 | 34.5 | 0.1 |
| **R Ulnar - Digit V (Antidromic) 140 mm** | | | | | | | | | |
| Wrist | Dig V | 3.0 | 4.0 | **4.2** | Wrist - Dig V | 140 | 46 | 34.7 | 0.1 |
|  |  |  |  |  | A.Elbow - Wrist |  |  |  |  |
| **R Radial - Anatomical snuff box (Forearm) 100 mm** | | | | | | | | | |
| Forearm | Wrist | 1.7 | 2.5 | **4.6** | Forearm - Wrist | 100 | 60 | 32.6 | 0.1 |
| **R Sural - Ankle (Calf)** | | | | | | | | | |
| Calf | Ankle | NR | NR | **NR** | Calf - Ankle | 140 | NR | 33.8 | 0.1 |
| **R Superficial peroneal - Ankle 120 mm** | | | | | | | | | |
| Lat leg | Ankle | NR | NR | **NR** | Lat leg - Ankle | 120 | NR | 33.6 | 0.1 |

**MNC**

| **Nerve / Sites** | **Muscle** | **Latency** | **Amplitude** | **Rel Amp** | **Duration** | **Area** | **Segments** | **Distance** | **Lat Diff** | **Velocity** | **Temp.** |
| --- | --- | --- | --- | --- | --- | --- | --- | --- | --- | --- | --- |
|  |  | **ms** | **mV** | **%** | **ms** | **mVms** |  | **mm** | **ms** | **m/s** | **°C** |
| **R Median - APB** | | | | | | | | | | | |
| Wrist | APB | 3.6 | **2.9** | 100 | 8.2 | 15.3 | Wrist - APB | 80 |  |  | 34.7 |
| Elbow | APB | 8.5 | **2.7** | 93.5 | 8.4 | 14.4 | Elbow - Wrist | 240 | 4.9 | 49 | 34.7 |
| Axilla | APB | 10.4 | **2.8** | 103 | 8.6 | 14.9 | Axilla - Elbow | 100 | 1.9 | 52 | 34.7 |
| **R Ulnar - ADM** | | | | | | | | | | | |
| Wrist | ADM | **3.8** | **2.3** | 100 | 6.0 | 6.9 | Wrist - ADM | 80 |  |  | 33.1 |
| B.Elbow | ADM | 7.7 | **2.2** | 95.9 | 5.6 | 6.3 | B.Elbow - Wrist | 200 | 4.0 | 51 | 33 |
| A.Elbow | ADM | 9.5 | **2.2** | 99 | 5.6 | 6.0 | A.Elbow - B.Elbow | 100 | 1.7 | 57 | 33 |
| Axilla | ADM | 11.4 | **2.1** | 96.1 | 5.5 | 6.1 | Axilla - A.Elbow | 100 | 1.9 | 53 | 32.9 |
| **R Peroneal - EDB** | | | | | | | | | | | |
| Ankle | EDB | NR | **NR** | NR | NR | NR | Ankle - EDB | 80 |  |  | 33.6 |
| **R Peroneal - Tib Ant** | | | | | | | | | | | |
| Fib Head | Tib Ant | 4.0 | **0.2** | 100 | 13.8 | 2.1 | Fib Head - Tib Ant |  |  |  | 33.6 |
| Pop fossa | Tib Ant | NR | **NR** | NR | NR | NR | Pop fossa - Fib Head |  | NR |  | 33.5 |
| **R Tibial - AH** | | | | | | | | | | | |
| Ankle | AH | NR | **NR** | NR | NR | NR | Ankle - AH | 80 |  |  | 33.6 |

**F Wave**

| **Nerve** | **F Lat** | **M Lat** |
| --- | --- | --- |
|  | **ms** | **ms** |
| **R Median - APB** | **N** | 3.6 |
| **R Ulnar - ADM** | **29.6** | 4.2 |

|  |  |  |  |  |  |  |  |  |  |
| --- | --- | --- | --- | --- | --- | --- | --- | --- | --- |
| **EMG Summary Table** |  | | | | | | | | |
|  | **Spontaneous** | | **Volitional MUAPs** | | | | **Max Vol Act** | | **-** |
| **Muscle** | **Fib/PSW** | **Fasc** | **Dur.** | **Amp** | **Poly** | **Recruit** | **Interference** | **Max Freq** | **Comments** |
| **R. Tibialis anterior** | **3+** | None |  |  |  |  |  |  | no MUAPs |
| **R. Gastrocnemius (Medial head)** | **3+** | None |  |  |  |  |  |  | no MUAPs |
| **R. Vastus medialis** | **3+** | None |  |  |  |  |  |  | no MUAPs |
| **R. First dorsal interosseous** | **2+** | None | **14-20** | 1.0-2.0 | **Many** | **Mod red** | Mod dec | 20 Hx | suprasegmental |
| **R. Triceps brachii** | **1+** | None | 8-12 | 0.4-1.2 | None | Normal | Full | 40 Hz | **crd** |
| **R. Deltoid** | **1+** | None | 8-12 | 0.4-1.2 | None | Normal | Full | 40 Hz | **abundant crds** |

**Supplementary Table 1. Nerve Conduction Studies and Electromyography results**. The NCS demonstrated: 1) reduced amplitude of the right median, ulnar, and peroneal-TA compound muscle action potentials (CMAPs), 2) absent right peroneal-EDB and tibial CMAPs, 3) reduced amplitude of the right median, ulnar, and radial sensory nerve action potentials (SNAPs), 4) absent right sural and superficial peroneal SNAPs, 5) a borderline increase in right ulnar distal motor latency, 6) borderline increase in the right ulnar minimum F-wave latencies and absent right median F-waves. The results of the EMG studies were abnormal for: 1) a high density of fibrillation potentials in the right tibialis anterior, gastrocnemius (medial head) and vastus medialis muscles and low to moderate density of fibrillation potentials in the right first dorsal interosseous, triceps and deltoid muscles, 2) complex repetitive discharges in the right triceps and deltoid muscles, 3) an increased incidence of long duration motor unit action potentials (MUAPs) in the right first dorsal interosseous muscle, 4) reduced recruitment of MUAPs right first dorsal interosseous muscle, and 5) no voluntary MUAPs seen in the right tibialis anterior, gastrocnemius (medial head), and vastus medialis muscles.
